# Sirolimus induces depletion of intracellular calcium stores and mitochondrial dysfunction in pancreatic beta cells

**DOI:** 10.1038/s41598-017-15283-y

**Published:** 2017-11-20

**Authors:** Angela Lombardi, Jessica Gambardella, Xue-Liang Du, Daniela Sorriento, Maurizio Mauro, Guido Iaccarino, Bruno Trimarco, Gaetano Santulli

**Affiliations:** 1 Department of Medicine, Albert Einstein College of Medicine, New York, NY USA; 2 Department of Advanced Biomedical Sciences, “Federico II” University of Naples, Naples, Italy; 3 Department of Medicine, Surgery and Dentistry, “Scuola Medica Salernitana”, University of Salerno, Salerno, Italy

## Abstract

Sirolimus (rapamycin) is an immunosuppressive drug used in transplantation. One of its major side effects is the increased risk of diabetes mellitus; however, the exact mechanisms underlying such association have not been elucidated. Here we show that sirolimus impairs glucose-stimulated insulin secretion both in human and murine pancreatic islets and in clonal β cells in a dose- and time-dependent manner. Importantly, we demonstrate that sirolimus markedly depletes calcium (Ca^2+^) content in the endoplasmic reticulum and significantly decreases glucose-stimulated mitochondrial Ca^2+^ uptake. Crucially, the reduced mitochondrial Ca^2+^ uptake is mirrored by a significant impairment in mitochondrial respiration. Taken together, our findings indicate that sirolimus causes depletion of intracellular Ca^2+^ stores and alters mitochondrial fitness, eventually leading to decreased insulin release. Our results provide a novel molecular mechanism underlying the increased incidence of diabetes mellitus in patients treated with this drug.

## Introduction

Post-transplant diabetes mellitus represents a major adverse effect of immunosuppressive drugs^1–4^ and is associated with high cumulative incidence of cardiac events, vascular disease, and overall impaired survival rates^5^. Sirolimus (rapamycin) was introduced in the Edmonton immunosuppression protocol in islet transplant recipients^6,7^, attempting to minimize the diabetogenic effects observed with corticosteroids and other immunosuppressive regimens. Despite the initial enthusiasm, 5-year results of this clinical trial revealed that only ~10% of patients maintained insulin independence^1,8^, endorsing the detrimental role of sirolimus in glucose homeostasis.

A randomized trial of immunosuppressive drugs in kidney transplantation, the Efficacy Limiting Toxicity Elimination (ELITE) – Symphony study^9^, identified sirolimus as the one with the highest incidence of hyperglycemia, even higher than calcineurin inhibitors^9^. Since then, several investigators sought to determine the mechanisms underlying new-onset diabetes mellitus after transplantation^10–13^. The effects of sirolimus *in vivo* are quite complex, as confirmed by numerous controversial findings: indeed, albeit several studies demonstrate that its administration causes glucose intolerance^14–16^, there are also reports showing that it does improve insulin sensitivity in diabetic mice^17^, protects against obesity^18,19^, reduces atherosclerosis^20,21^ and cardiac or renal fibrosis^22,23^, and extends lifespan^24^.

We decided to test the effects of sirolimus in pancreatic β cells. Our hypothesis is that one of the mechanisms underlying the diabetogenic action of sirolimus is the impairment of metabolism-secretion coupling in β cells. We focused on the effect of sirolimus on the key organelle in metabolism-secretion coupling, *i.e*. the mitochondrion^25–28^. Indeed, such organelle is considered the main responsible for coupling different fuel secretagogues to insulin exocytosis, through a process that includes oxidation of nutrients within the mitochondrial matrix and subsequent ATP generation, increasing intracellular calcium (Ca^2+^) via closure of ATP-sensitive K^+^ channels and depolarization of the plasma membrane^27–32^.

## Results

### Sirolimus impairs glucose-induced insulin secretion in pancreatic β cells

**Figure 1:**
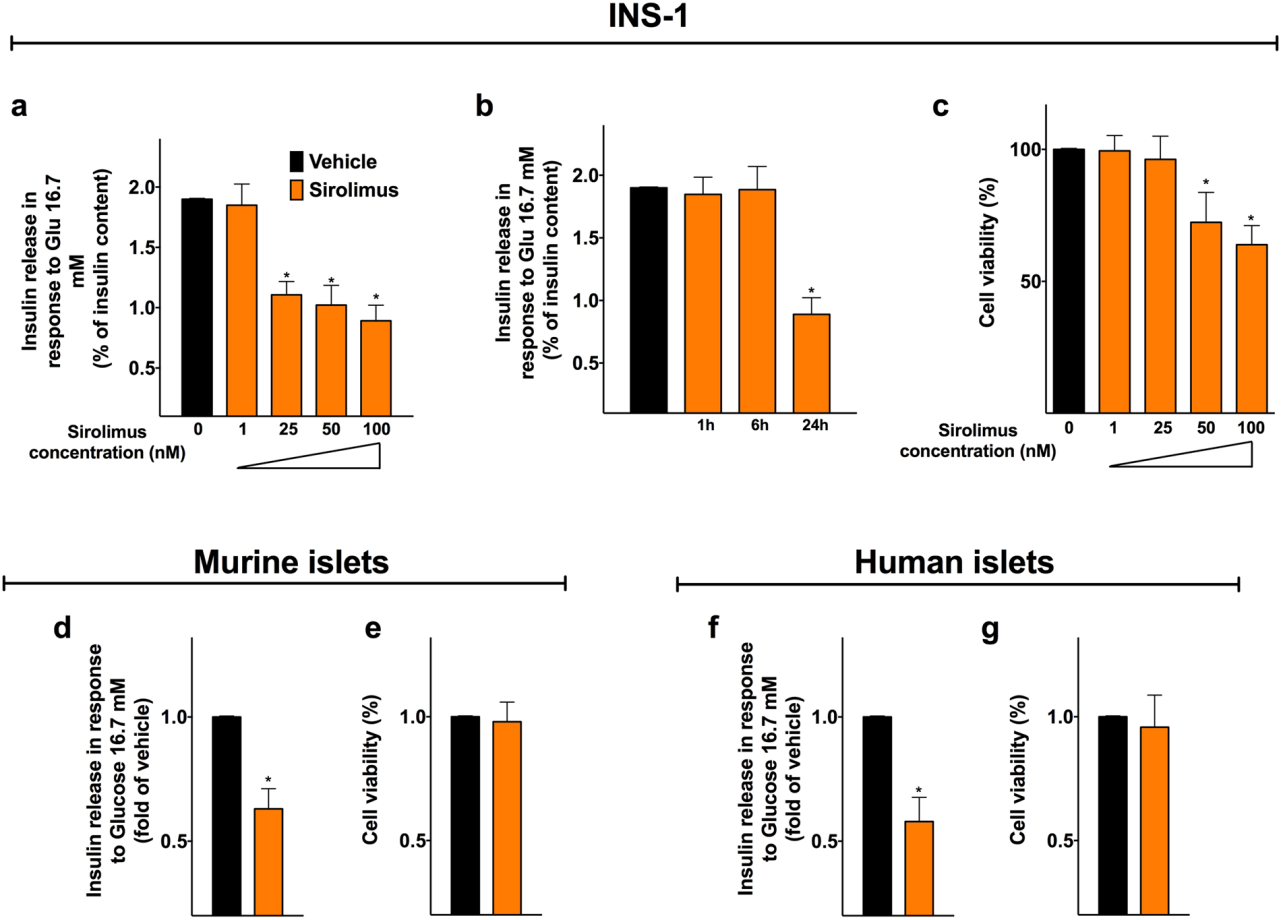
Sirolimus impairs glucose-stimulated insulin secretion from pancreatic β cells. Evaluation of the effect of sirolimus on clonal rat β cells (**a**–**c**), murine islets (**d**,**e**) and human islets (**f**,**g**). INS-1 β cells were treated for 24 h with vehicle or sirolimus at the indicated doses (**a**). INS-1 β cells were treated with vehicle or sirolimus (25 nM) for the indicated times (**b**). INS-1 β cells were treated for 24 h with 25 nM sirolimus (**c**). Effect of 25 nM sirolimus (24 h) on insulin release and cell viability in murine (**d**,**e**) and human islets (**f**,**g**). Data are presented as mean ± s.e.m of at least 5 experiments (clonal β cells and murine islets) or at least 3 experiments (human islets) performed in triplicate. *p < 0.05 *vs* vehicle. In panel c, data are expressed as percentage of the responses determined following treatment with vehicle, taken as 100%.

To test the effect of sirolimus on pancreatic β cell function, we evaluated the response to glucose in INS-1 β cells. We first performed a dose-response assay, and we found that increasing doses of sirolimus progressively reduce glucose-stimulated insulin secretion (GSIS, Fig. 1a). Then, we performed a time-course experiment using the dose of sirolimus (25 nM) that has been measured in the blood of transplant recipients^33^ and we observed that a 24-hour incubation significantly decreased GSIS (Fig. 1b). Importantly, we did not detect any significant effect of sirolimus on cell viability (Fig. 1c). These results were also confirmed in murine (Fig. 1d,e) and in human (Fig. 1f,g) islets.

### Sirolimus reduces mitochondrial respiration in pancreatic β cells

**Figure 2:**
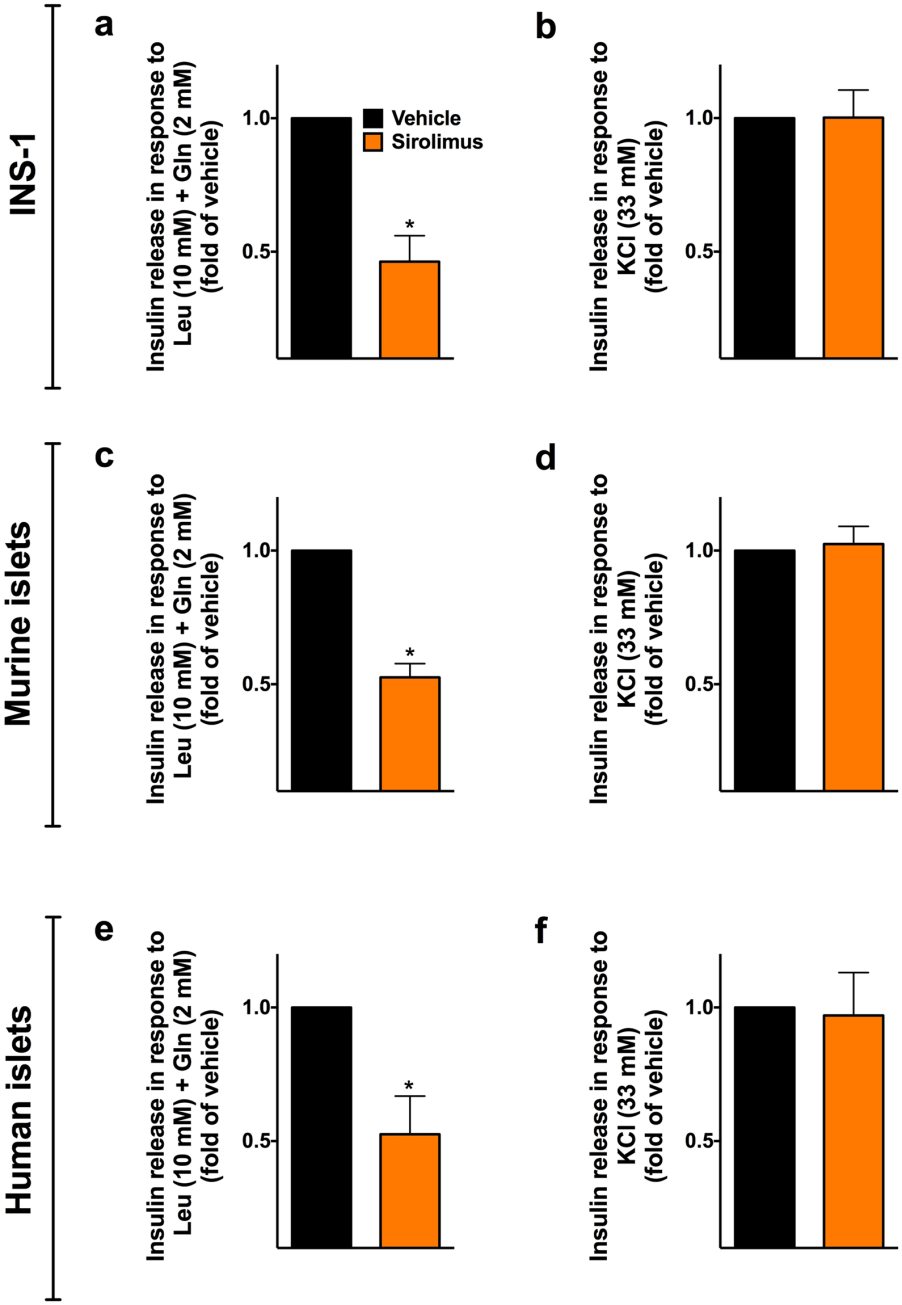
Sirolimus compromises insulin secretion from β cells in response to fuel secretagogues. INS-1 β cells (**a**,**b**), murine islets (**c**,**d**) and human islets (**e**,**f**) were incubated for 24 h with vehicle or 25 nM sirolimus and then stimulated with leucine (Leu) and glutamine (Gln, panels **a**,**c**,**e**) or with KCl (panels **b**,**d**,**f**). Data are presented as mean ± s.e.m of at least 3 experiments performed in triplicate. *p < 0.05 *vs* vehicle.

**Figure 3:**
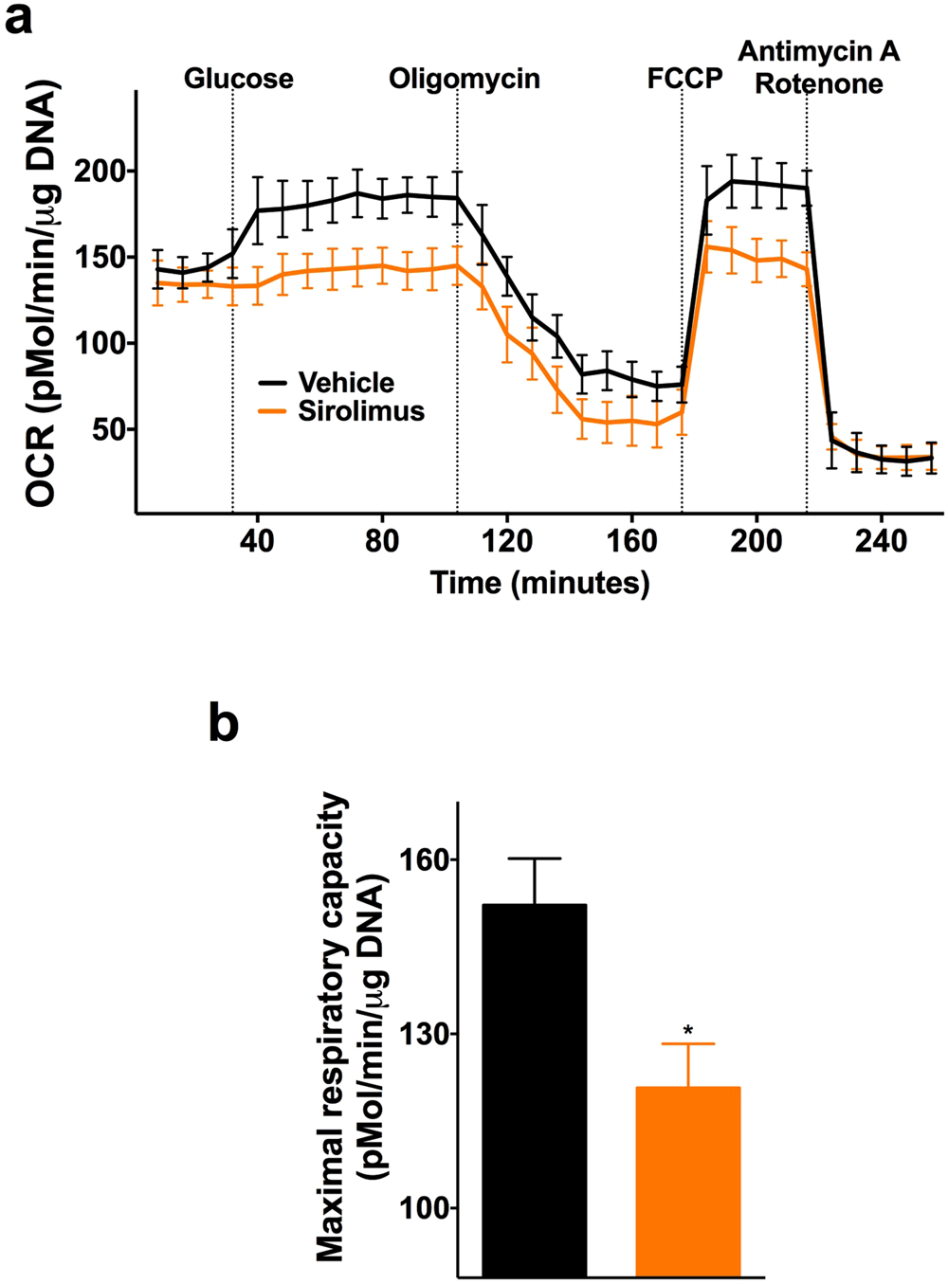
Sirolimus impairs mitochondrial respiration in pancreatic β cells. The time course of oxygen consumption rate (OCR) was measured using the Extracellular Flux Analyzer in β cells incubated for 24 h with vehicle or 25 nM sirolimus and then treated with glucose, oligomycin, phenylhydrazone (FCCP), antimycin A and rotenone (panel **a**); see methods for further details. The maximal respiratory capacity is quantified in panel **b**, in which whiskers represent 5% to 95% spread of the data. Data represent mean ± s.e.m. of 4 independent experiments, each performed in at least 7 replicates. *p < 0.05 *vs* vehicle.

When testing the effect of sirolimus on insulin release in response to the fuel secretagogues leucine and glutamine, which are known to stimulate insulin exocytosis through increased mitochondrial metabolism^34^, we found a significantly impaired response in sirolimus-treated cells (Fig. 2a), whereas cells from both groups were similarly responsive to KCl-mediated depolarization (Fig. 2b). We obtained similar findings in murine (Fig. 2c,d) and in human (Fig. 1e,f) islets, thereby suggesting an action of the immunosuppressant drug on the mechanisms underlying metabolism-secretion coupling. Therefore, we tested the effect of sirolimus on mitochondrial respiration, observing a significant decrease in oxygen consumption rate (OCR) in clonal β cells (Fig. 3) and in islets isolated from mice and humans (Figure S1) treated with sirolimus.

### Sirolimus decreases mitochondrial Ca^2+^ uptake in β cells

**Figure 4:**
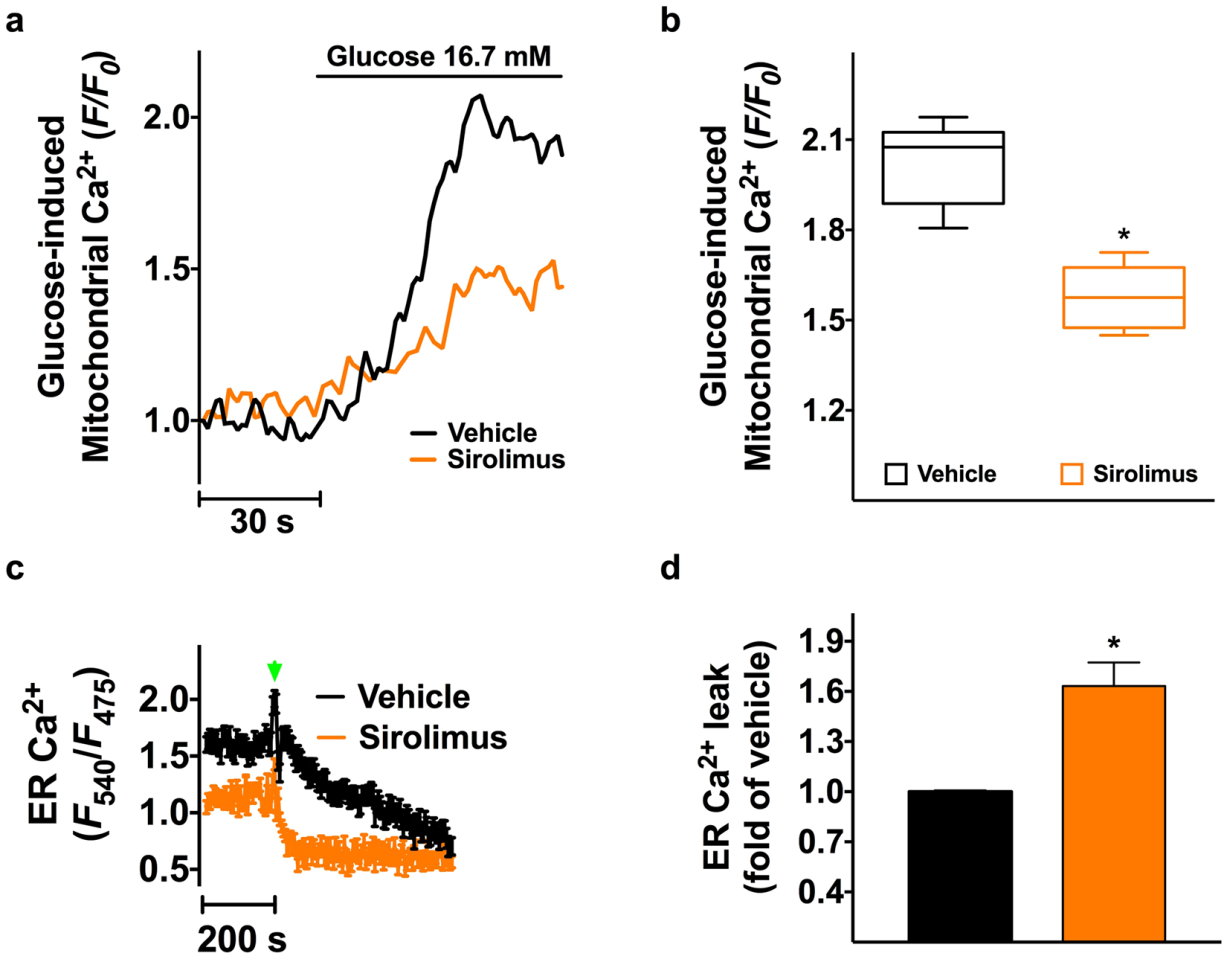
Effects of sirolimus on Ca^2+^ dynamics in mitochondria and endoplasmic reticulum (ER). Mitochondrial Ca^2+^ uptake was measured in clonal β cells incubated for 24 h with vehicle or 25 nM sirolimus and then stimulated with glucose (16.7 mM, panel **a**). Amplitude of mitochondrial response was calculated as the level of Rhod-2 *F*1/*F*_0_ at the peak (**b**). ER Ca^2+^ stores (**c**) and ER Ca^2+^ leak (**d**) were assessed following 24 h incubation with vehicle or 25 nM sirolimus. In panel c, the green arrowhead indicates thapsigargin (1 μm). Data are presented as mean ± s.e.m of at least 4 experiments performed in triplicate. *p < 0.05 *vs* vehicle. In panel b, whiskers represent 5% to 95% spread of the data.

Mounting evidence indicates that Ca^2+^ represents a major regulator of mitochondrial function, and a decreased uptake of this ion by this organelle has been functionally linked to reduced mitochondrial respiration in various cell types^35–39^. Thus, we assessed mitochondrial Ca^2+^ uptake in pancreatic β cells following incubation with sirolimus and we observed a significantly decreased uptake compared with vehicle-treated cells (Fig. 4a,b). We and others have demonstrated the importance of ER Ca^2+^ in β cell function^31,40–45^. Since sirolimus has been reported to modulate intracellular Ca^2+^ fluxes in different tissues^46–49^, we assessed ER Ca^2+^ stores and we found that sirolimus-treated β cells exhibited depleted intracellular Ca^2+^ stores and increased Ca^2+^ leak (Fig. 4c,d). When measuring cytosolic Ca^2+^ levels, we also observed a slightly reduced response to glucose in sirolimus-treated β cells (Figure S2), further supporting our data on decreased GSIS.

### Sirolimus regulates the expression of inositol 1,4,5-trisphosphate receptor in clonal β cells and human and murine islets

**Figure 5:**
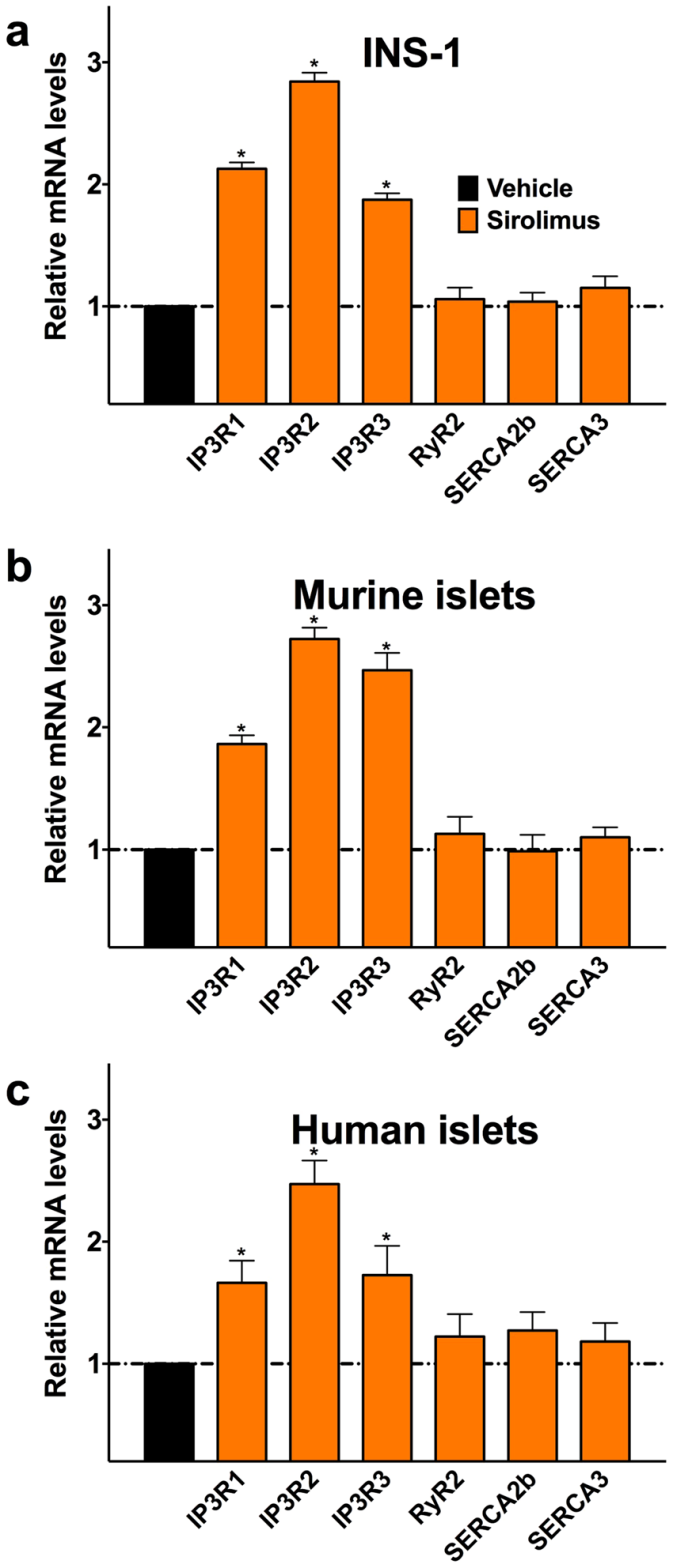
Effects of sirolimus on the expression of IP3Rs, RyR2 and SERCA in clonal β cells and murine and human islets. The effects of sirolimus (25 nM, 24 h) on mRNA levels of IP3Rs, RyR2, and SERCA in rat β cells (**a**) and murine (**b**) and human (**c**) islets were evaluated by real-time RT-qPCR analysis of total RNA, relative to vehicle-treated samples (horizontal dashed line), using GAPDH as internal standard. Primer sequences are reported in Supplementary Table 2. Each bar represents mean ± s.e.m. of at least 3 independent experiments in each of which reactions were performed in triplicate. *P < 0.05 vs vehicle.

Since sirolimus is known to modulate transcriptional activity^50–52^, we tested its effect on the expression levels of key players in Ca^2+^ handling, namely inositol 1,4,5-trisphosphate receptor (IP3R)^53^, ryanodine receptor (RyR)^31,54^ and sarco/endoplasmic reticulum Ca^2+^-ATPase (SERCA)^44^ in clonal β cells and human and murine islets. We observed a significant upregulation of all three IP3R isoforms in sirolimus-treated compared with vehicle-treated cells, a result that was consistent in all of the tested species (rat, mouse, human, Fig. 5), strongly suggesting that the modulation of IP3R is one of the mechanisms underlying the effects of sirolimus on pancreatic β cells.

## Discussion

Immunosuppressive therapy has been shown to be associated with glucose intolerance and post-transplantation diabetes mellitus; such a diabetogenic effect is common to immune-modulating agents acting via different mechanisms of action^3,9,55^, including β cell failure^4,13,56,57^ and alteration of metabolic parameters controlled by insulin signaling^58–61^. Here we show that sirolimus has a detrimental effect on Ca^2+^ handling and GSIS both in human and murine islets and in rat insulinoma cell line INS-1.

We also demonstrate that at 25 nM – the dose measured in the blood of transplanted patients^33^ – sirolimus does not significantly affect cell vitality, consistent with previous experiments performed in various cell types revealing that only supra-therapeutic doses of sirolimus lead to increased apoptosis and reduced cell proliferation^49,56,62^, without affecting overall insulin content^56^. Furthermore, earlier investigations had established that immunosuppressant drugs, when used at concentrations comparable with therapeutic levels in humans, do not cause apoptosis in pancreatic β cells^63^.

We show here for the first time that sirolimus significantly compromises mitochondrial respiration, directly assessed by measuring oxygen consumption, both in pancreatic islets and clonal β cells. The direct modulation of intracellular Ca^2+^ release channels on the ER offers a novel mechanistic insight on the diabetogenic effect of sirolimus. Intriguingly, the upregulation of IP3R observed in pancreatic β cells following sirolimus treatment is consistent with the recent observation that IP3R levels are increased in islets from diabetic patients, mirrored by a reduced number of interactions between ER and mitochondria^64^. Additionally, Madec and colleagues had shown that exposing pancreatic islets to high glucose concentrations led to increased levels of IP3R^65^. Also, mutations in the gene encoding for IP3R have been associated to perturbations in glucose homeostasis and enhanced susceptibility to diet-induced diabetes mellitus^66^. Further studies are necessary to better delineate the exact role of IP3Rs in the regulation of Ca^2+^ fluxes in β cells and to identify other potential mechanisms.

Interestingly, the properties of sirolimus observed in β cells are cell-specific and seem to be in contrast with its effects seen in models of neurodegenerative and ischemic disorders^67,68^, in which the drug has been shown to be overall protective, inducing autophagy and enhancing lysosomal activation in order to remove damaged mitochondria^69^. The complexity of the pathways induced by sirolimus is further confirmed by the experimental findings of Fuhrer and colleagues, who observed that, despite sirolimus significantly suppresses β cell response to glucose (in agreement with our findings), the incubation of RIN-5F cells with high doses of sirolimus in absence of glucose can instead increase insulin secretion^57^. However, opposite to their results, Barlow and colleagues found that 200 nM sirolimus caused a significant reduction in both basal and glucose-stimulated insulin release in Min-6 cells^56^. The exact mechanisms underlying such different pharmacologic responses need to be characterized in future studies.

## Materials and Methods

### Cells

Human islets with >90% purity and viability were obtained from non-diabetic de-identified cadaveric donors through the *Integrated Islet Distribution Program* (*IIDP*). The characteristics of the donors are reported in Supplementary Table 1. Upon receipt, the islets were cultured as described^70^. Murine islets of Langerhans were isolated as previously described^31^. Procedures on rodents have been performed according to guidelines and regulations approved by the Einstein Animal Care and Use Committee. INS-1 cells were maintained in monolayer culture in RPMI-1640 medium, as previously described by our group^71^. Insulin levels were determined as described and validated^31,71–73^. In some experiments the cells were treated with glucose (5.5 and 16.7 mM, Bio-Techne, Abingdon, UK), sirolimus (LC Laboratories, Woburn, MA, dissolved in dymethylsulfoxide), or L-leucine (10 mM, MyBioSource, San Diego, CA, USA) and glutamine (2 mM, MyBioSource). Cell viability was estimated by the [3-(4,5-dimethylthiazol-2-yl)-2,5-diphenyl tetrazolium bromide, MTT colorimetric assay, spectophotometrically (570 nm) measuring the ability of metabolically active cells to reduce MTT.

### Extracellular flux analyses

Extracellular flux analyses were performed using the Extracellular Flux Analyzer (Agilent Technologies, Santa Clara, CA, USA), according to the manufacturer’s instructions. Specifically, the following drugs were added to each well: glucose (16.7 mM, at minute 32) to determine response to high glucose; oligomycin (1 μM, at minute 104) to inhibit ATP synthase and assess coupling efficiency; carbonyl cyanide 4-(trifluoromethoxy) phenylhydrazone (FCCP, 0.5 μM, at minute 176) to uncouple the mitochondrial oxidative phosphorylation and measure both maximum respiration and spare capacity; antimycin A and rotenone (both 1 μM, at minute 216) to inhibit the respiratory chain and measure non-mitochondrial respiration. After each assay, the cells were collected to quantify DNA via QuantiFluor dsDNA System (Promega, Madison, WI, USA), according to the manufacturer’s instructions.

### Ca^2+^ dynamics in cytosol, ER and mitochondria

Ca^2+^ dynamics were evaluated as previously described and validated^74–77^. Briefly, cells attached on glass bottom culture dishes (MatTek Corporation, Ashland, MA) were loaded with Fura-2 acetoxymethyl (AM) ester (Thermo Fisher Scientific, Waltham, MA, USA, 5 μM, 15 min, 37 °C). Images were obtained using a dual excitation fluorescence imaging system, as described^31^: changes in intracellular Ca^2+^ were expressed as the ratio of fluorescence emission acquired above 510 nm in response to excitation at 340 nm and 380 nm. ER Ca^2+^ was assessed in cells transfected with the luminal Ca^2+^ sensor D1ER (Addgene, Cambridge, MA), as described^78–80^ and the rate of Ca^2+^ leak was measured as function of [Ca^2+^]_ER_ following the addition of thapsigargin (1 μM). To evaluate mitochondrial Ca^2+^, the samples were loaded with rhod-2 AM (Thermo Fisher Scientific, 3 μM, 30 min, 37 °C), followed by washout and 1 hour rest at room temperature for de-esterification^31,77,81^. Fluorescence was detected using a pass-band filter of 545–625 nm in response to excitation at 542 nm.

### Real-time RT-qPCR

Total RNA was isolated from β cells and islets using TRIzol reagent (Thermo Fisher Scientific) in combination with the RNeasy Mini kit (Qiagen, Hilden, Germany) followed by DNase treatment^82,83^, and cDNA was synthesized via a Thermo-Script RT-PCR System (Thermo Fisher Scientific). After reverse transcription, real-time quantitative PCR was performed on an AbiPRISM 7300 fast real-time cycler using the power SYBR Green real-time PCR master mix kit and quantified by built-in SYBR Green Analysis (Thermo Fisher Scientific)^77,84^. Samples were measured in triplicates and results were confirmed by at least three independent experiments. The relative amount of specific mRNA was normalized to Glyceraldehyde 3-phosphate dehydrogenase (*GAPDH*). The sequences of oligonucleotide primers (Merck KGaA, Darmstadt Germany) for gene analysis are listed in Supplementary Table 2.

### Statistical analysis

All results are presented as mean ± s.e.m. Unless otherwise noted, experiments were performed in a blinded fashion at least three times. Statistical analysis was performed via Student’s *t* test (for 2 groups) unless otherwise indicated, using Prism 7 software (GraphPad, San Diego, CA, USA). A value of *P* < 0.05 was considered statistically significant.

### Data Availability

All data generated or analyzed during this study are included in the present article.

## Electronic supplementary material

Supplementary Information
