# Supplementary material for: Sirolimus induces depletion of intracellular calcium stores and mitochondrial dysfunction in pancreatic beta cells
Source: Sci Rep. 2017 Nov 20;7:15823. doi: 10.1038/s41598-017-15283-y (PMC5696524; doi:10.1038/s41598-017-15283-y)
Supplement: Supplementary file 1 — Supplementary Information [file 41598_2017_15283_MOESM1_ESM.pdf]

Sirolimus induces depletion of intracellular calcium stores and mitochondrial dysfunction in pancreatic beta cells

Angela Lombardi, Jessica Gambardella, Xue-Liang Du, Daniela Sorriento, Maurizio Mauro, Guido Iaccarino, Bruno Trimarco, Gaetano Santulli

Supplementary info:

2 Supplementary Figures

2 Supplementary Tables

Supplementary Figures

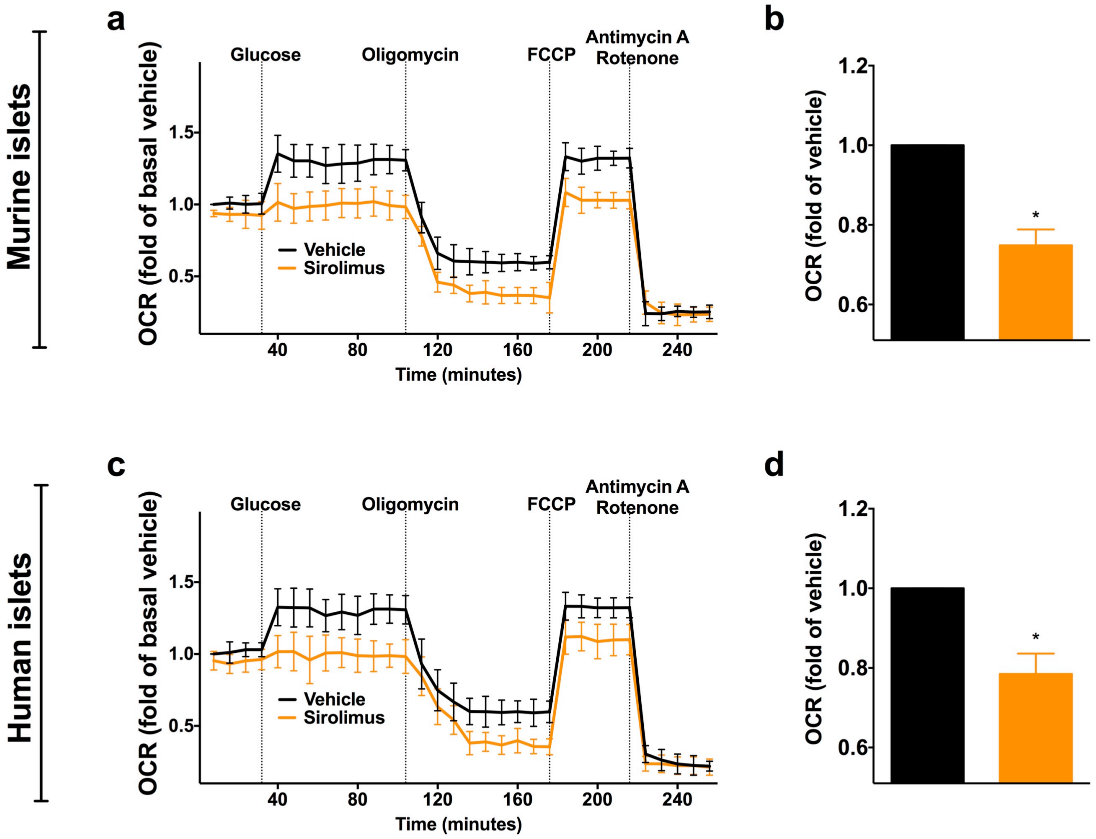

Supplementary Figure 1.

Sirolimus impairs mitochondrial respiration in murine and human islets.

Oxygen consumption rate (OCR) was measured using the Extracellular Flux Analyzer in murine (**a,b**) and human (**c,d**) islets incubated for 24h with vehicle or 25 nM sirolimus and then treated with glucose, oligomycin, phenylhydrazone (FCCP), antimycin A, and rotenone. The maximal respiratory capacity is quantified in panels **b** and **d**. Data are presented as mean ± s.e.m. of 3 independent experiments. *:$p<0.05$ *vs* vehicle.

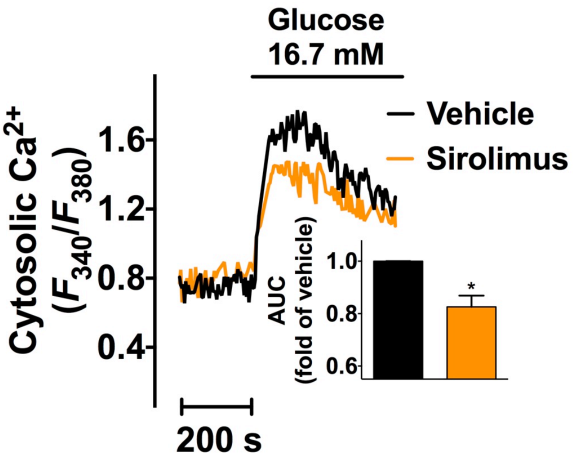

Supplementary Figure 2.

Effects of sirolimus on cytosolic Ca^{2+} dynamics in clonal β cells.

Representative curves of cytosolic Ca^{2+} dynamics in INS-1 β cells loaded with Fura-2 AM in response to glucose 16.7 mM. **Inset**: Area under curve (AUC); data are presented as mean±s.e.m of 5 independent experiments performed in triplicate. *:p<0.05 *vs* vehicle.

Supplementary Table 1– Characteristics of human islet donors

Donor	Age	Sex	BMI (kg/m^2)
**1**	27	M	30
**2**	61	M	27
**3**	30	M	25.9
**4**	45	M	36.4

BMI: Body mass index.

Supplementary Table 2 – Sequences of oligonucleotide primers and product sizes (in bp)

	Human	Mouse	Rat
**IP3R1**	GGGTTCAAGAGGGAATGGGA	CGGCTGCTCTCTCATTTGTC	ACCTTCTCCTCCCATTGGTG
	CCATGTCCCCACTTCTCTGT	CTTGGTTAGCTGAGGTGGGA	CATCCGAAGAGTGCACATGG
	100	95	90
**IP3R2**	ACCTGGATTGCTGTGCTAGT	TCTCAGCTGTCCTCTCCTCT	CAGCGAATCTCTTCCGTGTG
	AAAAGATCCCTCTGGCTGCT	AGAGCAACTGGGAGGAGAAC	TGCATTGTGGGTAGGGTCAT
	102	107	103
**IP3R3**	GAGAAGTCAGAGCTGTGGGT	CTACACAGACATGCGGCTTC	AGGAATGGGTCTTCTGTGGG
	GGAGCCCCACTTACCTCTTT	TCTGACTTCCCCGTGGTAAC	ACTTTCTCCCTGCCTCTTCC
	98	110	111
**RyR2**	CCTTGCCTGAGTGCAGTTG	TTCCCCAAGATGGTGACAAG	ACTTATGAGCACAGCAGGGT
	TTGAGGTATCAACAGGTTGTGG	CTCCAGCAGGTAGCTCAGGT	AGGGAATTTGGGGAGGAAGG
	130	105	100
**SERCA2b**	TCGTTATGTTCAGCCCAGGT	CATGTCTAGCAGAACACGGC	AATTGTCGTAGGTGCATCGC
	CATGCCTGTAATCCCAGCAC	CACCACACTTAACCTGGCAC	AGCCAGCAGAGACAGGAAAT
	91	96	98
**SERCA3**	AAGGAAGGAGGCAGCATCTT	AGACAGACCTGACCTTCGTG	GCTTGTGTTCCCGTTTCTGT
	TGAAGGACCCAATTCTCCCC	CATGACCACTCGAATGCCAG	GGCCCAAAAGTGAGAAGGTG
	111	107	103
**GAPDH**	GGGACTGGCTTTCCCATAAT	CACTGAGCATCTCCCTCACA	TCTCCCTCACAATTCCATCC
	TGTGGTCTGCAAAAGGAGTG	GTGGGTGCAGCGAACTTTAT	AGAGGGTGCAGCGAACTTTA
	94	111	99

References

1. Shapiro, A.M., *et al.* International trial of the Edmonton protocol for islet transplantation. *N Engl J Med* **355**, 1318-1330 (2006).
2. Krentz, A.J. & Wheeler, D.C. New-onset diabetes after transplantation: a threat to graft and patient survival. *Lancet* **365**, 640-642 (2005).
3. Shivaswamy, V., Boerner, B. & Larsen, J. Post-Transplant Diabetes Mellitus: Causes, Treatment, and Impact on Outcomes. *Endocr Rev* **37**, 37-61 (2016).
4. D'Amico, E., Hui, H., Khoury, N., Di Mario, U. & Perfetti, R. Pancreatic beta-cells expressing GLP-1 are resistant to the toxic effects of immunosuppressive drugs. *J Mol Endocrinol* **34**, 377-390 (2005).
5. Li, L.C., *et al.* Proteinuria and baseline renal function predict mortality and renal outcomes after sirolimus therapy in liver transplantation recipients. *BMC Gastroenterol* **17**, 58 (2017).
6. Shapiro, A.M., *et al.* Islet transplantation in seven patients with type 1 diabetes mellitus using a glucocorticoid-free immunosuppressive regimen. *N Engl J Med* **343**, 230-238 (2000).
7. Manning, B.D. Game of TOR - The Target of Rapamycin Rules Four Kingdoms. *N Engl J Med* (2017).
8. Ryan, E.A., *et al.* Five-year follow-up after clinical islet transplantation. *Diabetes* **54**, 2060-2069 (2005).
9. Ekberg, H., *et al.* Reduced exposure to calcineurin inhibitors in renal transplantation. *N Engl J Med* **357**, 2562-2575 (2007).
10. Lamming, D.W., *et al.* Rapamycin-induced insulin resistance is mediated by mTORC2 loss and uncoupled from longevity. *Science* **335**, 1638-1643 (2012).
11. Hjelmesaeth, J., Midtvedt, K., Jenssen, T. & Hartmann, A. Insulin resistance after renal transplantation: impact of immunosuppressive and antihypertensive therapy. *Diabetes Care* **24**, 2121-2126 (2001).
12. Houde, V.P., *et al.* Chronic rapamycin treatment causes glucose intolerance and hyperlipidemia by upregulating hepatic gluconeogenesis and impairing lipid deposition in adipose tissue. *Diabetes* **59**, 1338-1348 (2010).
13. Paty, B.W., Harmon, J.S., Marsh, C.L. & Robertson, R.P. Inhibitory effects of immunosuppressive drugs on insulin secretion from HIT-T15 cells and Wistar rat islets. *Transplantation* **73**, 353-357 (2002).
14. Whiting, P.H., *et al.* Toxicity of rapamycin--a comparative and combination study with cyclosporine at immunotherapeutic dosage in the rat. *Transplantation* **52**, 203-208 (1991).
15. Zhang, N., *et al.* Sirolimus is associated with reduced islet engraftment and impaired beta-cell function. *Diabetes* **55**, 2429-2436 (2006).
16. Yang, S.B., *et al.* Rapamycin induces glucose intolerance in mice by reducing islet mass, insulin content, and insulin sensitivity. *J Mol Med (Berl)* **90**, 575-585 (2012).
17. Deepa, S.S., *et al.* Rapamycin Modulates Markers of Mitochondrial Biogenesis and Fatty Acid Oxidation in the Adipose Tissue of db/db Mice. *J Biochem Pharmacol Res* **1**, 114-123 (2013).
18. Chang, G.R., *et al.* Rapamycin protects against high fat diet-induced obesity in C57BL/6J mice. *J Pharmacol Sci* **109**, 496-503 (2009).
19. Yang, S.B., *et al.* Rapamycin ameliorates age-dependent obesity associated with increased mTOR signaling in hypothalamic POMC neurons. *Neuron* **75**, 425-436 (2012).
20. Castro, C., *et al.* Rapamycin attenuates atherosclerosis induced by dietary cholesterol in apolipoprotein-deficient mice through a p27 Kip1 -independent pathway. *Atherosclerosis* **172**, 31-38 (2004).

21. Chen, W.Q., *et al.* Oral rapamycin attenuates inflammation and enhances stability of atherosclerotic plaques in rabbits independent of serum lipid levels. *Br J Pharmacol* **156**, 941-951 (2009).
22. Haller, S.T., *et al.* Rapamycin Attenuates Cardiac Fibrosis in Experimental Uremic Cardiomyopathy by Reducing Marinobufagenin Levels and Inhibiting Downstream Pro-Fibrotic Signaling. *J Am Heart Assoc* **5**(2016).
23. Rafehi, H. & El-Osta, A. HDAC Inhibition in Vascular Endothelial Cells Regulates the Expression of ncRNAs. *Non-Coding RNA* **2**, 4 (2016).
24. Kennedy, B.K. & Lamming, D.W. The Mechanistic Target of Rapamycin: The Grand ConducTOR of Metabolism and Aging. *Cell Metab* **23**, 990-1003 (2016).
25. Gauthier, B.R., *et al.* PDX1 deficiency causes mitochondrial dysfunction and defective insulin secretion through TFAM suppression. *Cell Metab* **10**, 110-118 (2009).
26. Akhmedov, D., *et al.* Mitochondrial matrix pH controls oxidative phosphorylation and metabolism-secretion coupling in INS-1E clonal beta cells. *FASEB J* **24**, 4613-4626 (2010).
27. Kennedy, E.D. & Wollheim, C.B. Role of mitochondrial calcium in metabolism-secretion coupling in nutrient-stimulated insulin release. *Diabetes Metab* **24**, 15-24 (1998).
28. Fujimoto, S., *et al.* Impaired metabolism-secretion coupling in pancreatic beta-cells: role of determinants of mitochondrial ATP production. *Diabetes Res Clin Pract* **77 Suppl 1**, S2-10 (2007).
29. Aizawa, T. & Komatsu, M. Rab27a: a new face in beta cell metabolism-secretion coupling. *The Journal of clinical investigation* **115**, 227-230 (2005).
30. Wang, H., Gauthier, B.R., Hagenfeldt-Johansson, K.A., Iezzi, M. & Wollheim, C.B. Foxa2 (HNF3beta) controls multiple genes implicated in metabolism-secretion coupling of glucose-induced insulin release. *The Journal of biological chemistry* **277**, 17564-17570 (2002).
31. Santulli, G., *et al.* Calcium release channel RyR2 regulates insulin release and glucose homeostasis. *The Journal of clinical investigation* **125**, 1968-1978 (2015).
32. Hughes, S.J., *et al.* Electrophysiological and metabolic characterization of single beta-cells and islets from diabetic GK rats. *Diabetes* **47**, 73-81 (1998).
33. Desai, N.M., *et al.* Elevated portal vein drug levels of sirolimus and tacrolimus in islet transplant recipients: local immunosuppression or islet toxicity? *Transplantation* **76**, 1623-1625 (2003).
34. Vetterli, L., *et al.* Delineation of glutamate pathways and secretory responses in pancreatic islets with beta-cell-specific abrogation of the glutamate dehydrogenase. *Molecular biology of the cell* **23**, 3851-3862 (2012).
35. Perocchi, F., *et al.* MICU1 encodes a mitochondrial EF hand protein required for Ca(2+) uptake. *Nature* **467**, 291-296 (2010).
36. Naghdi, S., *et al.* Mitochondrial Ca2+ uptake and not mitochondrial motility is required for STIM1-Orai1-dependent store-operated Ca2+ entry. *J Cell Sci* **123**, 2553-2564 (2010).
37. Fu, A., *et al.* LKB1 couples glucose metabolism to insulin secretion in mice. *Diabetologia* **58**, 1513-1522 (2015).
38. Santulli, G. & Marks, A.R. Essential roles of intracellular calcium release channels in muscle, brain, metabolism, and aging. *Current Molecular Pharmacology* **8**, 206-222 (2015).
39. Bononi, A., *et al.* BAP1 regulates IP3R3-mediated Ca2+ flux to mitochondria suppressing cell transformation. *Nature* **546**, 549-553 (2017).
40. Clark, A.L., *et al.* Targeting Cellular Calcium Homeostasis to Prevent Cytokine-Mediated Beta Cell Death. *Scientific reports* **7**, 5611 (2017).
41. Rutter, G.A., *et al.* Local and regional control of calcium dynamics in the pancreatic islet. *Diabetes Obes Metab* **19 Suppl 1**, 30-41 (2017).
42. Kang, G., *et al.* A cAMP and Ca2+ coincidence detector in support of Ca2+-induced Ca2+ release in mouse pancreatic beta cells. *The Journal of physiology* **566**, 173-188 (2005).

43. Gwiazda, K.S., Yang, T.L., Lin, Y. & Johnson, J.D. Effects of palmitate on ER and cytosolic Ca2+ homeostasis in beta-cells. *Am J Physiol Endocrinol Metab* **296**, E690-701 (2009).
44. Tong, X.*, et al.* SERCA2 Deficiency Impairs Pancreatic beta-Cell Function in Response to Diet-Induced Obesity. *Diabetes* **65**, 3039-3052 (2016).
45. Bertram, R., Sherman, A. & Satin, L.S. Electrical bursting, calcium oscillations, and synchronization of pancreatic islets. *Adv Exp Med Biol* **654**, 261-279 (2010).
46. Luik, R.M., Wang, B., Prakriya, M., Wu, M.M. & Lewis, R.S. Oligomerization of STIM1 couples ER calcium depletion to CRAC channel activation. *Nature* **454**, 538-542 (2008).
47. Lamming, D.W., Ye, L., Sabatini, D.M. & Baur, J.A. Rapalogs and mTOR inhibitors as anti-aging therapeutics. *J Clin Invest* **123**, 980-989 (2013).
48. Santulli, G. & Totary-Jain, H. Tailoring mTOR-based therapy: molecular evidence and clinical challenges. *Pharmacogenomics* **14**, 1517-1526 (2013).
49. Li, J., Kim, S.G. & Blenis, J. Rapamycin: one drug, many effects. *Cell Metab* **19**, 373-379 (2014).
50. Fielhaber, J.A.*, et al.* Inactivation of mammalian target of rapamycin increases STAT1 nuclear content and transcriptional activity in alpha4- and protein phosphatase 2A-dependent fashion. *The Journal of biological chemistry* **284**, 24341-24353 (2009).
51. Wang, Y.*, et al.* Regulation of androgen receptor transcriptional activity by rapamycin in prostate cancer cell proliferation and survival. *Oncogene* **27**, 7106-7117 (2008).
52. Laberge, R.M.*, et al.* MTOR regulates the pro-tumorigenic senescence-associated secretory phenotype by promoting IL1A translation. *Nat Cell Biol* **17**, 1049-1061 (2015).
53. Luciani, D.S.*, et al.* Roles of IP3R and RyR Ca2+ channels in endoplasmic reticulum stress and beta-cell death. *Diabetes* **58**, 422-432 (2009).
54. Blodgett, D.M.*, et al.* Novel Observations From Next-Generation RNA Sequencing of Highly Purified Human Adult and Fetal Islet Cell Subsets. *Diabetes* **64**, 3172-3181 (2015).
55. Han, E., Kim, M.S., Kim, Y.S. & Kang, E.S. Risk assessment and management of post-transplant diabetes mellitus. *Metabolism* **65**, 1559-1569 (2016).
56. Barlow, A.D.*, et al.* Rapamycin toxicity in MIN6 cells and rat and human islets is mediated by the inhibition of mTOR complex 2 (mTORC2). *Diabetologia* **55**, 1355-1365 (2012).
57. Fuhrer, D.K., Kobayashi, M. & Jiang, H. Insulin release and suppression by tacrolimus, rapamycin and cyclosporin A are through regulation of the ATP-sensitive potassium channel. *Diabetes Obes Metab* **3**, 393-402 (2001).
58. Bianchi, G., Marchesini, G., Marzocchi, R., Pinna, A.D. & Zoli, M. Metabolic syndrome in liver transplantation: relation to etiology and immunosuppression. *Liver Transpl* **14**, 1648-1654 (2008).
59. Nagaraja, P., Ravindran, V., Morris-Stiff, G. & Baboolal, K. Role of insulin resistance indices in predicting new-onset diabetes after kidney transplantation. *Transpl Int* **26**, 273-280 (2013).
60. Wyzgal, J.*, et al.* Insulin resistance in kidney allograft recipients treated with calcineurin inhibitors. *Ann Transplant* **12**, 26-29 (2007).
61. Bogan, J.S. Endocytic cycling of glucose transporters and insulin resistance due to immunosuppressive agents. *J Clin Endocrinol Metab* **99**, 3622-3624 (2014).
62. Van de Velde, S., Hogan, M.F. & Montminy, M. mTOR links incretin signaling to HIF induction in pancreatic beta cells. *Proceedings of the National Academy of Sciences of the United States of America* **108**, 16876-16882 (2011).
63. Hernandez-Fisac, I.*, et al.* Tacrolimus-induced diabetes in rats courses with suppressed insulin gene expression in pancreatic islets. *Am J Transplant* **7**, 2455-2462 (2007).
64. Thivolet, C., Vial, G., Cassel, R., Rieusset, J. & Madec, A.M. Reduction of endoplasmic reticulum-mitochondria interactions in beta cells from patients with type 2 diabetes. *PLoS One* **12**, e0182027 (2017).

65. Madec, A.M., *et al.* Losartan, an angiotensin II type 1 receptor blocker, protects human islets from glucotoxicity through the phospholipase C pathway. *FASEB J* **27**, 5122-5130 (2013).
66. Ye, R., *et al.* Inositol 1,4,5-trisphosphate receptor 1 mutation perturbs glucose homeostasis and enhances susceptibility to diet-induced diabetes. *J Endocrinol* **210**, 209-217 (2011).
67. Bove, J., Martinez-Vicente, M. & Vila, M. Fighting neurodegeneration with rapamycin: mechanistic insights. *Nat Rev Neurosci* **12**, 437-452 (2011).
68. Li, Q., *et al.* Rapamycin attenuates mitochondrial dysfunction via activation of mitophagy in experimental ischemic stroke. *Biochemical and biophysical research communications* **444**, 182-188 (2014).
69. Galluzzi, L., *et al.* Molecular definitions of autophagy and related processes. *EMBO J* (2017).
70. Lombardi, A. & Tomer, Y. Interferon alpha impairs insulin production in human beta cells via endoplasmic reticulum stress. *J Autoimmun* **80**, 48-55 (2017).
71. Santulli, G., *et al.* Age-related impairment in insulin release: the essential role of beta(2)-adrenergic receptor. *Diabetes* **61**, 692-701 (2012).
72. Lombardi, A., *et al.* Increased hexosamine biosynthetic pathway flux dedifferentiates INS-1E cells and murine islets by an extracellular signal-regulated kinase (ERK)1/2-mediated signal transmission pathway. *Diabetologia* **55**, 141-153 (2012).
73. Fiory, F., *et al.* Methylglyoxal impairs insulin signalling and insulin action on glucose-induced insulin secretion in the pancreatic beta cell line INS-1E. *Diabetologia* **54**, 2941-2952 (2011).
74. Xie, W., *et al.* Imaging atrial arrhythmic intracellular calcium in intact heart. *J Mol Cell Cardiol* **64**, 120-123 (2013).
75. Umanskaya, A., *et al.* Genetically enhancing mitochondrial antioxidant activity improves muscle function in aging. *Proc Natl Acad Sci U S A* **111**, 15250-15255 (2014).
76. Gambardella, J., Trimarco, B., Iaccarino, G. & Santulli, G. New Insights in Cardiac Calcium Handling and Excitation-Contraction Coupling. *Adv Exp Med Biol* (2017).
77. Santulli, G., Xie, W., Reiken, S.R. & Marks, A.R. Mitochondrial calcium overload is a key determinant in heart failure. *Proceedings of the National Academy of Sciences of the United States of America* **112**, 11389-11394 (2015).
78. Palmer, A.E., Jin, C., Reed, J.C. & Tsien, R.Y. Bcl-2-mediated alterations in endoplasmic reticulum Ca2+ analyzed with an improved genetically encoded fluorescent sensor. *Proceedings of the National Academy of Sciences of the United States of America* **101**, 17404-17409 (2004).
79. Carmosino, M., *et al.* The expression of Lamin A mutant R321X leads to endoplasmic reticulum stress with aberrant Ca2+ handling. *J Cell Mol Med* **20**, 2194-2207 (2016).
80. Yang, Y.H., Manning Fox, J.E., Zhang, K.L., MacDonald, P.E. & Johnson, J.D. Intraislet SLIT-ROBO signaling is required for beta-cell survival and potentiates insulin secretion. *Proceedings of the National Academy of Sciences of the United States of America* **110**, 16480-16485 (2013).
81. Xie, W., *et al.* Mitochondrial oxidative stress promotes atrial fibrillation. *Sci Rep* **5**, 11427 (2015).
82. Santulli, G., *et al.* A selective microRNA-based strategy inhibits restenosis while preserving endothelial function. *J Clin Invest* **124**, 4102-4114 (2014).
83. Lombardi, A., Inabnet, W.B., 3rd, Owen, R., Farenholtz, K.E. & Tomer, Y. Endoplasmic reticulum stress as a novel mechanism in amiodarone-induced destructive thyroiditis. *J Clin Endocrinol Metab* **100**, E1-10 (2015).
84. Sorriento, D., *et al.* Intracardiac injection of AdGRK5-NT reduces left ventricular hypertrophy by inhibiting NF-kappaB-dependent hypertrophic gene expression. *Hypertension* **56**, 696-704 (2010).
